# Characteristics of Interface Zone Between Glass-Based Restorative Materials and Sound and Caries-Affected Primary Dentine

**DOI:** 10.3390/ma19030568

**Published:** 2026-02-02

**Authors:** Jelena Vulovic, Vukasin Kosutic, Sanja Kojic, Lazar Milic, Jovana Kuzmanovic Pficer, Bojan Petrovic, Aleksandar Racic, Marko Zivkovic, Tamara Peric

**Affiliations:** 1 School of Dental Medicine, University of Belgrade, Dr Subotica 8, 11000 Belgrade, Serbia; 2020.0119@stomf.bg.ac.rs (V.K.); 2019.4007@stomf.bg.ac.rs (A.R.);; 2 Department of Power, Electronics and Telecommunication, Faculty of Technical Sciences, University of Novi Sad, Trg Dositeja Obradovica 6, 21000 Novi Sad, Serbia; 3 Department for Medical Statistics and Informatics, School of Dental Medicine, University of Belgrade, Dr Subotica 1, 11000 Belgrade, Serbia; jovana.kuzmanovic@stomf.bg.ac.rs; 4 Dentistry Clinic of Vojvodina, Faculty of Medicine, University of Novi Sad, Hajduk Veljkova 3, 21000 Novi Sad, Serbia; bojan.petrovic@mf.uns.ac.rs; 5 Clinic of Pediatric and Preventive Dentistry, School of Dental Medicine, University of Belgrade, Dr Subotica 11, 11000 Belgrade, Serbia

**Keywords:** glass hybrid cement, glass ionomer cements, primary dentin, artificial caries-affected dentin, adhesive interface, microleakage, SEM

## Abstract

The aim was to evaluate the interface zone between glass hybrid (GH), high-viscosity (HV), and resin-modified (RM) glass-ionomer cements (GIC) and sound (SD) and artificially created caries-affected (ACAD) primary dentin. Occlusal cavities were prepared in 120 extracted primary molars and randomly assigned to SD or ACAD. Samples were restored with GH (Equia Forte HT-EF), HV-GIC (Equia Fill-E; Ketac Molar-KM), or RM-GIC (Fuji-II LC-FII; Photac Fill-PF) and exposed to thermal aging. Microleakage (methylene-blue) was analyzed using an optical digital microscope. The interface between dental tissues and restorative materials was analyzed using a scanning electron (SE) microscope. SE microphotographs were manually annotated for intimate contact and cracks/gaps at the material-enamel/dentin interface and analyzed using a custom Python-based algorithm to quantify the respective percentages. No microleakage was noted only in the SD group for EF (50%), FII (42%), and E (33%). All materials showed higher microleakage in ACAD than SD (*p* < 0.05). No continuous intimate contact between restorative material and dental tissues was observed along the entire interface. The mean proportion of intimate contact between the material and SD was EF (76%) > KM (55%) > E (38%) > FII (7%) > PF (4%), and EF (32%) > KM (24%) > E (16%) > FII (15%) > PF (0%) for ACAD (*p* < 0.05). Caries-induced demineralization affects the quality of the material–dentin interface. GH are likely to provide better sealing compared to the previous generations of GI materials.

## 1. Introduction

Dental treatment of pediatric dental patients encompasses various behavioral and clinical challenges. Children often exhibit limited attention spans, are easily distracted, and may have a reduced cooperation during dental treatment [1]. The behavioral challenges are further complicated by practical limitations, such as difficulties in maintaining a dry working field and the need for fast, efficient interventions.

Glass ionomer cements (GIC) are commonly applied in pediatric dental practice to address many clinical challenges due to ease of handling, tolerance to moisture, sustained fluoride release, and potential anticariogenic effects of the materials [2,3]. However, despite the advantages, the longevity of GIC restorations is limited by lower bond strength and wear resistance compared to resin composites [4].

Material performances may vary significantly between the formulations. Conventional GIC (C-GIC), while bioactive, demonstrate increased failure rates in multi-surface restorations of primary teeth, largely attributed to their mechanical properties [5,6]. Resin-modified GIC (RM-GIC) demonstrate improved clinical performances, showing survival rates comparable to compomers and composite resins in multi-surface restorations in primary molars [7].

A milestone in the clinical application of restorative GIC was the development of high-viscosity (HV-) GIC with multiple modifications that enhanced the performances of the material: incorporation of polyacrylic acid into the powder in dry form, increased molecular weight of the acid in the liquid component, and a higher powder-to-liquid ratio [8]. These modifications increase secondary cross-linking, resulting in improved mechanical properties compared to earlier C-GIC formulations. HV-GIC exhibit significantly greater flexural strength, reducing the risk of cohesive failure, previously recognized as a principal cause of restoration failure in C-GIC [8].

Glass hybrid (GH) materials incorporate ultrafine, highly-reactive glass particles, in addition to conventional fluoro-alumino-silicate particles found in C-GIC [9], thereby increasing the available reactive surface area per unit volume and enhancing the potential for chemical bonding [10]. Moreover, the polyacrylic acid used in both powder and liquid components of GH materials is of higher molecular weight, which increases cross-linking within the matrix and results in enhanced mechanical properties [10]. Moshaverinia et al. [11] reported higher compressive strength and microhardness for GHC compared to C-GIC.

Restorative materials are applied to various substrates under clinical conditions. Carious dentin is composed of two distinct layers with distinct morphological, biochemical, bacteriological, and physiological properties. The outer layer shows irreversible alteration, with extensive demineralization, denatured collagen fibers, and bacterial infection. The inner carious dentin is reversibly demineralized, retains an intact collagen network, is not infected, and has the potential for remineralization [12]. Clinically, the gold standard is to remove the infected and necrotic layer to stop lesion progression and ensure adequate support for the restoration, while preserving the affected but remineralizable dentin to maintain tooth integrity [13]. Therefore, restorative materials must adhere to various substrates, including inner, caries-affected dentin.

The quality of the material–tooth interface is an important determinant of restoration success. It reflects the mechanical and structural continuity between the restorative material and dental substrate and has direct implications for the integrity and durability of the restoration. A well-formed, continuous interface contributes to uniform stress distribution and reduces the likelihood of debonding or crack initiation under functional loads. Conversely, poor material adaptation or gaps at the interface can act as initiation points for cracks or debonding and may facilitate penetration of oral fluids and bacteria and consequently reactivation of caries process [14,15]. Moreover, examining the tooth-material interface provides valuable insights into the interactions between the restorative material and the underlying dental tissues.

The aim of this study was to evaluate characteristics of the interface zone between sound (SD) and artificially created caries-affected (ACAD) primary dentin and three types of restorative materials: GH, C-GIC, and RM-GIC. The null hypothesis was that no significant differences in microleakage and interface zone characteristics would be found between the materials when applied to SD and ACAD.

## 2. Materials and Methods

Ethical approval for the study was obtained from the local ethics committee (Document 36/1 issued on 7 March 2022). One hundred and twenty intact human primary molars were used. Teeth were extracted close to exfoliation in children who had difficulties with chewing, experienced discomfort/pain due to loosening and soft tissue impingement, or orthodontic reasons. The study procedures were explained to both the children and their parents/guardians prior to tooth extraction. Written informed consent was secured from the parent/guardian, while verbal assent was provided by the children.

**Figure 1:**
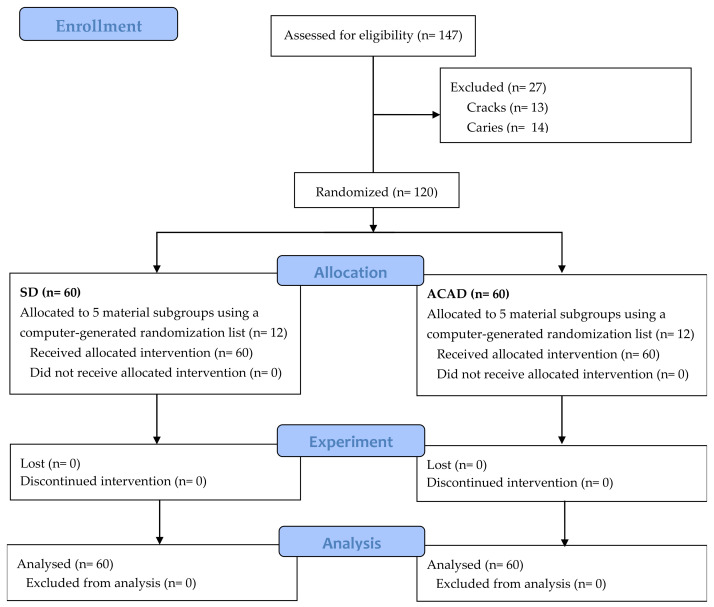
Workflow of the study.

The study workflow is summarized in Figure 1. Soft debris and periodontal ligaments were removed from the teeth after extraction. Each tooth was examined for cracks, hypomineralization, and caries. The selected teeth were autoclaved at 121 °C (Vacuklav 24B+, MELAG, Berlin, Germany) for 20 min to ensure infection control, stored in physiological saline at +4 °C, and used no later than a month after extraction.

All experimental procedures were performed by a single operator. Occlusal cavities 4 × 3 × 2 mm [16] were prepared using a water-cooled diamond bur (ISO 806 314 173 524 016, Diaswiss S.A., Nyon, Switzerland). The cavity depth was ensured by placing a rubber stopper on the bur. Teeth were placed in silicone molds (Zetaplus, Zhermack SpA, Rome, Italy) to ensure that the cavity floor is parallel to the floor. The samples were randomized into the two groups: (1) SD and (2) ACAD: a demineralizing solution (1.5 mM CaCl_2_, 0.9 mM KH_2_PO_4_, and 50 mM acetic acid adjusted to pH 4.5 with NaOH), was applied with a pipette into the prepared cavities to a maximum depth of 1 mm for 7 days at room temperature [17]. Cavities were inspected every 12 h for evaporation of the demineralising solution, which was replenished when required.

Samples were randomly allocated into subgroups (*n* = 12) using a computer-generated randomization list and restored with a GHC (Equia Forte HT, GC Int., Tokyo, Japan- EF), 2 HV-GIC (Equia Fill, GC Int., Tokyo, Japan- E; Ketac Molar, 3M ESPE, St. Paul, MN, USA- KM), and 2 RM-GIC (Fuji II LC, GC Int., Tokyo, Japan- F-II, Photac Fill, 3M ESPE, St. Paul, MN, USA- PF). Cavity restoration was performed according to manufacturers’ instructions (Table 1).

After the material had set, the samples were immersed in artificial saliva (30 mM/L KCl, 1.2 mM/L CaCl_2_, and 0.72 mM/L KH_2_PO_4_, adjusted to pH 7.0 with KOH) [18], for 7 days at 37 °C (CIBAC45 cooling circulating bath, COLO, Novo Mesto, Slovenia). To age, samples were subjected to 10,000 thermal cycles in aqueous media at temperatures of 5–55 °C, with a dwell time of 30 s and 1-second intervals between baths [19].

**Figure 2:**
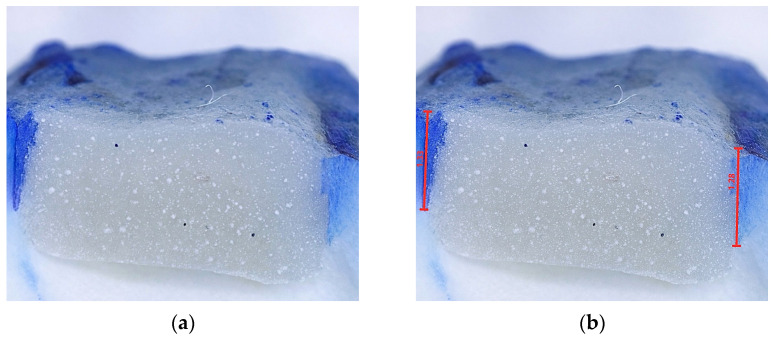
Microleakage evaluation using a digital optical microscope (**a**) before and (**b**) after image annotation. Dye penetration was measured in millimeters.

The apical portion of the tooth was closed using a composite material (Gradia posterior, GC Int.) to prevent the penetration of the dye into the root canal system and the pulp chamber of the tooth. The tooth surfaces were coated with a nail polish up to 1 mm from the restoration margins. The teeth were immersed in 1% methylene blue for 24 h [20]. Teeth were thoroughly rinsed with water and cut using a water-cooled circular saw (Isomet Low-Speed Saw, Buehler, Lake Bluff, IL, USA) in the inciso-gingival direction, through the central fissure [21]. Microleakage was analyzed at two points (in the area of the mesial and distal cavity walls), using a digital optical microscope (5M 300× USB Digital Microscope, Mustech Electronics Co., Shenzhen, China) at 40× magnification, using MicroCapture Pro software version 3.0 (Figure 2) and averaged for each sample. Optical microscopy and microleakage annotations were performed by a trained operator who was blinded to the experimental procedures. A second operator independently reviewed the annotated images to ensure inter-examiner agreement.

The interface between dental tissues and restorative materials was analyzed using a scanning electron (SE) microscope (SEM, HITACHI TM3030, Hitachi Ltd., Tokyo, Japan) under a 15 kV accelerating voltage. The imaging was conducted at a constant magnification of ×40, selected to provide an optimal field of view for capturing the material–tooth interface and sufficient spatial resolution to show cracks and flaws. The working distance remained constant, ranging from 6.7 mm to 8.5 mm, contingent upon the size of the item being scanned. All images were captured in backscattered electron (BSE) composition mode, enhancing compositional contrast and facilitating the unambiguous observation of structural discontinuities. The image size was configured to 1280 × 1100 pixels (grayscale), and the brightness and contrast parameters were uniform across all photographs captured to eliminate bias in post-processing. The imaging was conducted in a low-vacuum setting, with the scan speed configured at Slow3 to minimize noise and enhance edge clarity. This ensured consistent imaging settings and a uniform signal across all samples.

**Figure 3:**
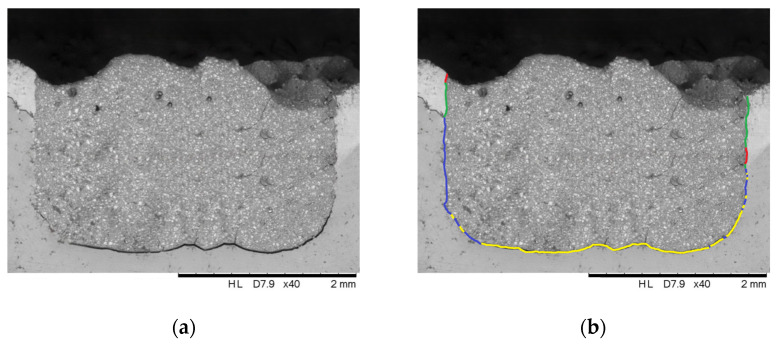
SE micrograph of the material–tooth interface (**a**) before and (**b**) after marking as follows: green, intimate contact between the material and enamel; red, crack at the material–enamel interface; blue, intimate contact between the material and dentin; yellow, crack at the material–dentin interface.

One image per sample was annotated using Microsoft Paint software version 11.2402.32.0 to mark the intimate contact of the material and hard tooth tissues or cracks at the material-dental tissue interface. Intimate contact and cracks/gaps were classified qualitatively as either continuous or intermittent along the material-tooth interface. Intimate contact was defined as adequate adhesion between the restorative material and the tooth tissue and was annotated in blue for dentin and green for enamel. A crack/gap was defined as an irregular void between the restorative material and the tooth tissue and was annotated in yellow for dentin and red for enamel (Figure 3). Initially, three operators, blinded to the experimental procedures, independently assessed all SE microphotographs. One operator was then trained using a single group of materials. Following this training phase, the remaining two operators reviewed and evaluated the annotations performed during training. Once consensus and consistency were achieved, the trained operator annotated all remaining groups. Finally, the other two operators independently assessed the annotated images to validate the annotations and ensure inter-observer reliability.

**Figure 4:**
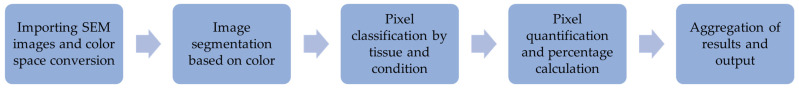
Flowchart of the proposed algorithm.

Following color annotation, a color-overlay-driven workflow was implemented in the Python programming language, 3.12 PyCharm Community Editiok environment, through OpenCV and Numpy. Each SEM image was processed in its entirety, without cropping or grayscale normalization. After loading the image, it was converted from their native RGB color space to the HSV color space to ensure robust separation of the annotation colors from the grayscale background. Segmentation was then performed by fixed HSV thresholding to generate binary masks corresponding to four predefined classes: green and red for “good” and “bad” features in enamel, and blue and yellow for “good” and “bad” features in dentin. Red features were detected using two hue intervals to account for hue wrap-around in HSV space. The algorithm subsequently quantified the number of pixels belonging to each color class ($N_{pixels\;in\;dentin}=N_{pixels\;in\;blue\;}+N_{pixels\;in\;yellow}$) and calculated their relative proportions (as percentages) with respect to the total-colored pixel count. Finally, the results from all processed images are aggregated and exported in a structured output CSV format (Figure 4).

The sample size calculation was derived according to a previous study of microtensile bond strength [22]. One-way ANOVA with fixed effects was used to compare the differences between the five mean values (five materials) at a significance level of 0.05 and a study power of 95%. As a result, a sample size of minimum 20 specimens for each material was established, effectively allocating 10 specimens per subgroup. Due to the possibility of dropouts during the study, 24 specimens per materials were included. In addition, a post hoc power analysis performed for the microleakage for five materials achieved statistical power of 85.7% for SD (ANOVA: fixed effects, omnibus, one-way; α = 0.05; dz = 0.4981, N = 60) and 91.6% for ACAD (ANOVA: fixed effects, omnibus, one-way; α = 0.05; dz = 0.5427 N = 60). Sample size calculations and post hoc power analyses were conducted using G*Power software version 3.1.9.4 (Düsseldorf, Germany).

Statistical analyses were performed using SPSS Statistics version 22 (IBM Corp., Armonk, New York, NY, USA). Nonparametric data were described using the median and interquartile range (IQR), and categorical data were described using percentages. Normality of data distribution was assessed using the Shapiro–Wilk test. For comparisons involving more than three groups, the Kruskal–Wallis test was initially performed. If the result was significant, pairwise comparisons were made using the Mann–Whitney U test, with the Holm correction applied to control for multiple comparisons. The Holm correction was applied in the R software package version 4.5.1 (Düsseldorf, Germany) to control for Type I error in multiple testing. Aligned-rank transform (ART) analysis was performed in the R software package version 4.5.1 using ARTool package version 0.11.2. This allowed testing the effects of material, dentin type, and their interaction. Post hoc pairwise comparisons were conducted with Bonferroni adjustment (emmeans package version 2.0.1). Effect sizes were reported using partial eta-squared (η^2^) interpreted according to Cohen [23] as small (η^2^ = 0.01), medium (η^2^ = 0.06), and large (η^2^ = 0.14) effects. Categorical data were analyzed using the chi-square test. Statistical significance was set at *p* < 0.05.

## 3. Results

### 3.1. Microleakage

For both SD (*n* = 60, *p* < 0.001, Kruskal–Wallis test) and ACAD (*n* = 60, *p* < 0.001, Kruskal–Wallis test), microleakage differed significantly between the materials. The microleakage of the analyzed restorative materials was as follows:

- For SD: EF (0.19 (0.53) mm) < E (0.32 (0.58) mm) < F-II (0.33 (0.88) mm) < KM (0.69 (1.31) mm) < PF (0.97 (0.64) mm). PF showed significantly higher microleakage compared to EF, E (*n* = 24, *p* < 0.001, Mann–Whitney test), and F-II (*n* = 24, *p* < 0.05, Mann–Whitney test, Holm-corrected).
- For ACAD: E (0.52 (1.31) mm) < EF (0.73 (0.98) mm) < F-II (0.94 (0.22) mm) < PF (1.39 (0.32) mm) < KM (2.00 (1.01) mm). Microleakage differed significantly between KM and E, EF (*n* = 24, *p* < 0.001, Mann–Whitney test, Holm-corrected), and F-II (*n* = 24, *p* < 0.05, Mann–Whitney test, Holm-corrected), and between PF and EF and F-II (*n* = 24, *p* < 0.05, Mann–Whitney test, Holm-corrected).

Within the PF and KM groups, microleakage values differed significantly between SD and ACAD in favor of ACAD (*n* = 24, *p* < 0.05, Mann–Whitney test, Holm-corrected).

Due to exploring the interaction of two factors (material and dentin type), a nonparametric factorial analysis was performed using the aligned-rank transform (ART). The interaction between material and dentin type was not statistically significant (F (4,120)) = 1.28, *p* = 0.28, indicating that the effect of material was consistent across dentin types. The interaction between material and dentin (η^2^ = 0.0639) was small, suggesting minimal interaction between factors. Both material (F (4,120) = 16.25, *p*< 0.001, η^2^ = 0.5326) and dentin type (F (1,120)) = 43.19, *p* < 0.001, η^2^ = 0.4035) had a significant main effect on microleakage. Post hoc comparison indicated that PF and KM exhibited the highest values for microleakage. The bootstrap CIs for η^2^ for material were [0.45, 0.61] and for dentin were [0.35, 0.45]. Post hoc comparisons (Bonferroni-adjusted) revealed that PF and KM exhibited significantly higher values compared to EF, E, and F-II (*p* < 0.001), while no significant difference was observed between PF and KM.

**Figure 5:**
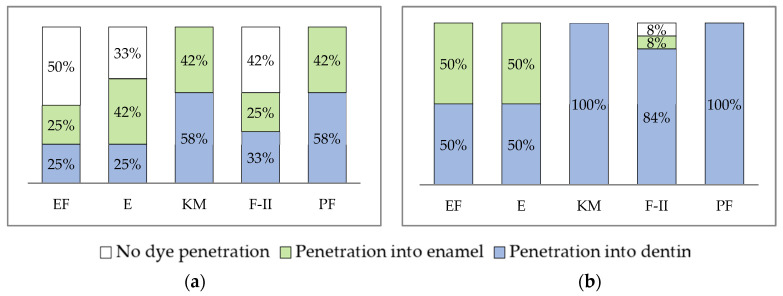
Microleakage in (**a**) SD, and (**b**) ACAD group. Equia Forte HT-EF; Equia Fill-E; Ketac Molar-KM; Fuji II-F-II; Photac Fill-PF.

**Figure 6:**
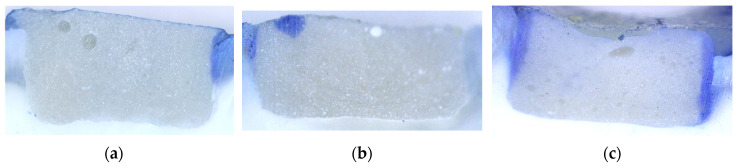
Representative microphotographs of a specimen showing (**a**) no microleakage, (**b**) dye penetration into enamel, and (**c**) dye penetration into dentin.

No microleakage was noted for EF (50%), F-II (42%), and E (33%) in the SD group (*p* < 0.05, chi-square test) and for F-II (8%) in ACAD. In both SD and ACAD, materials differed significantly in regard to depth of color penetration (*p* < 0.05, chi-square test; Figure 5). Representative microphotographs of specimens with various extent of dye penetration are shown in Figure 6.

### 3.2. SEM

**Figure 7:**
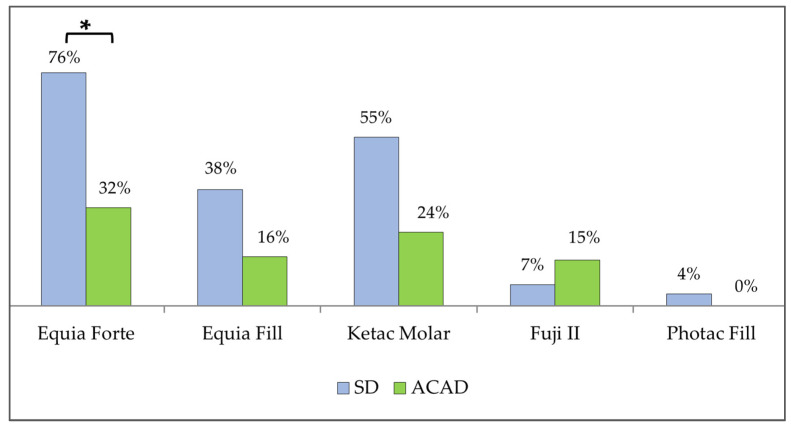
The mean proportion of intimate contact between the material and dentin. * *p* < 0.05 (Mann–Whitney test) between SD and ACAD.

No continuous intimate contact of the material and dental tissues along the entire interface was observed. GH demonstrated significantly better adherence to both SD and ACAD compared to C-GIC and RM-GIC (*p* < 0.05, Kruskal–Wallis test). In addition, GH adhered better to SD than to ACAD (*p* < 0.05, Mann–Whitney test; Figure 7).

A continuous crack along the entire material–tooth interface was observed in the C-GIC groups, in 33% of E and 17% of KM in the SD group, and in 33% of both E and KM samples in the ACAD group.

**Figure 8:**
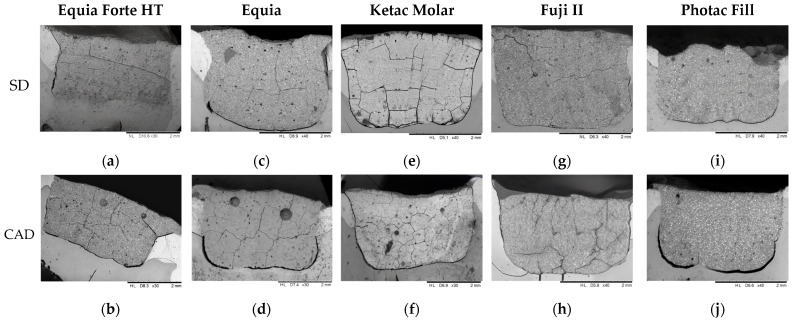
SE microphotographs of the material–tooth interface. The restorative materials exhibited a fine-grained microstructure with variable presence of voids within the matrix. (**a**) EF showing good adaptation to SD; (**b**) EF showing partial separation at the material–ACAD interface; (**c**) E showing partial separation at the material–SD interface; (**d**) E showing a more extensive gap at the material–ACAD interface; (**e**) evident gap at the KM-SD interface; (**f**) evident gap at the material–ACAD interface; (**g**) F-II showing partial adaptation to SD; (**h**) F-II showing partial separation to ACAD; (**i**) PF showing partial adaptation to SD; (**j**) PF showing larger areas of contact loss at the material–ACAD interface.

Representative SE microphotographs of the materials tested are shown in Figure 8.

## 4. Discussion

Modern concepts in dentistry have shifted focus away from traditional non-selective caries removal. Contemporary strategies emphasize minimally invasive techniques, which include selective removal of carious tissue [24,25]. Sealing CAD with a restorative material of sufficient integrity may impair the nutrient flow to remaining microorganisms, thereby creating unfavorable conditions for their survival. This will lead to arrestment of the lesion and protection of the pulpo-dentine complex [13]. Kuhn et al. [26] demonstrated biochemical changes within sealed carious dentin over 60 days, indicating a reduction in dentin degradation activity and early signs of tissue stabilization following sealing.

The oral environment is characterized by constant thermal, chemical, and mechanical stress that can influence the integrity of the restoration over time. GIC address these challenges through their unique bonding mechanism. This mechanism relies on an initial weak interaction with enamel and dentin that gradually matures into stable ionic bonding through carboxyl groups, providing durable adhesion [27]. A distinct ion-exchange layer develops at the tooth-GIC interface. Furthermore, their thermal expansion coefficients closely match those of natural tooth tissues, and unlike resin composites, they do not exhibit polymerization shrinkage [28]. These properties minimize microleakage and enhance marginal adaptation, critical for restoration longevity in the dynamic oral environment.

Microleakage testing can be a relevant indicator of the material sealing ability and a possible predictor of restoration failure [28]. While extensive research has evaluated microleakage in permanent teeth, studies in primary teeth remain limited, particularly regarding newer GIC and GH formulations. Among the tested materials, GH demonstrated the lowest microleakage in both substrates. Lower microleakage of GH compared to RM-GIC and composite materials has also been reported in permanent teeth [29,30]. The results of the present study confirm previous findings that HV-GIC accomplish superior marginal sealing compared to RM-GIC [31]. The higher microleakage of RM-GIC might be explained by the polymerization shrinkage associated with its resin component during light curing [32].

Since the experimental design involved two factors (material × substrate), ART analysis was employed to evaluate main effects and interactions within a nonparametric framework. Both material and dentin substrate showed significant effect on microleakage.

The present study revealed differences in microleakage when materials were applied to SD and ACAD. Alves et al. [31] reported that both HV-GIC and RM-GIC effectively bonded to CAD, with nanoleakage patterns comparable to those observed in SD. Marquezan et al. [33] reported reduced interfacial adaptation of RM-GIC in CAD relative to SD. Using micro-CT, Gaintantzopoulou et al. [34] also confirmed the superior adaptation of HV-GIC, which formed a more continuous and compact adaptation to cavity walls in primary teeth compared to RM-GIC, where more frequent interfacial defects were observed.

Variances in material application protocols, suggested by the manufacturers, may influence the longevity of the restorations. Proper cavity pretreatment with polyalkenoic acid plays a significant role in achieving optimal bonding efficacy of GIC to dentin [35]. The presence of a smear layer created during cavity preparation may interfere with bonding effectiveness by impeding the direct interaction of the restorative material with the dentin substrate [36]. In the present study, PF and KM exhibited the highest microleakage, which may be attributed to the absence of the cavity conditioning in PF groups, as well as the absence of a protective varnish for both materials, which plays a crucial role in sealing the restoration and preventing early moisture contamination. Thomas et al. [37] reported a significant reduction in occlusal margin microleakage when a coating was applied over a GIC restoration in primary teeth, indicating its role in enhancing the sealing ability. Similarly, Habib et al. [38] observed that nanocoats, particularly Equia Forte Coat, improve the physicomechanical properties and adaptation of GH and RM-GIC, indicating the clinical benefits of surface coating in optimizing the longevity and performance of GI restorative materials. Furthermore, Gürler et al. [36] demonstrated that GH adhered more effectively to dentin than C-GIC regardless of the conditioning protocol, which was attributed to their higher viscosity, improved mechanical properties, and the presence of a nanofilled surface coating that enhanced cavity wall adaptation.

To our knowledge, this is the first study to integrate algorithm-assisted quantitative SEM analysis, enabling objective comparison of interface quality across dentin substrate under standardized conditions, rather than relying solely on qualitative observations. Findings from the SEM analysis aligned well with those of the microleakage evaluation. GH demonstrated superior adaptation to dentin compared to HV-GIC and RM-GIC, which corresponded with its lower microleakage. However, cracks observed in SE microphotographs should be interpreted with caution, as they may reflect artifacts caused by brittleness of GI materials and the mechanical stress introduced during sample sectioning and preparation. The colored pixels used by the algorithm originated from manual annotation overlays, resulting in a human-in-the-loop, semi-quantitative approach rather than a fully automatic crack detection pipeline. The algorithm itself is deterministic: given the same annotated input image, it yields identical results. This workflow was selected to ensure transparency and reproducibility while allowing direct quantitative extraction of crack metrics from manually verified annotations. Potential sources of error are therefore limited to the consistency of manual annotation, color thresholding tolerance, and automatic scale-bar detection.

Although the results of the present study showed increased occurrence of interfacial gaps when materials were applied to ACAD, the ability of GI materials to maintain adaptation on demineralized substrates supports their clinical applicability in selective caries removal strategies. GIC form chemical bonds with hydroxyapatite calcium ions, allowing them to adhere effectively to both SD and partially demineralized dentin [27]. This bonding mechanism is not hindered by the presence of CAD, ensuring stable adhesion across different dentin substrates [22]. Furthermore, the sustained fluoride release of GIC and ion exchange with hard dental tissues might enhance their therapeutic role in arresting caries progression, promoting remineralization, and preserving dentin structure [39].

Limitations of the present study are acknowledged by the authors. The artificial induction of carious lesions provides a model for assessing restorative material performance on demineralized substrates. However, this in vitro approach does not replicate biological responses of dentin-pulp complex such as dentinal tubule occlusion with mineral contents [40]. On the other hand, artificially created carious dentin is more uniform than natural carious dentin, with a homogenized mineral content [17], thereby ensuring standardization of experimental procedures. Not including simulated masticatory loading is another limitation of the present study. Still, thermocycling realistically reproduces challenging conditions of the oral environment and the aging of the material. Temperature fluctuations from dietary hot/cold cycles create stress to the material, and unequal thermal expansion of restorative materials and tooth tissues promotes microgap formation [33]. Although concerns have been raised regarding a potential reduction in dentin microhardness following steam autoclaving, previous studies have demonstrated that this process does not result in significant alterations in dentin morphology or chemical composition [41], nor does it affect dentin permeability [42,43] or bond strength [42]. Accordingly, it can be expected that steam autoclaving does not compromise tooth structure or influence the subsequent analysis of restorative materials. In addition, microleakage and SEM, although providing valuable information about dentin-material interface, allow only two-dimensional surface analysis without deeper insights into the spatial morphology or internal structure of the interface. Nevertheless, analyzing interfaces using two-dimensional resources still offers high analytical precision in many contexts. Two-dimensional methods simplify complex spatial interactions, enable assessment of surface properties, morphological patterns, and interface relations, facilitating visualization, image processing, and quantitative analysis. Future studies employing three-dimensional imaging techniques, such as micro-computed tomography, may allow a more comprehensive volumetric evaluation of material adaptation and defect distribution at the tooth–material interface.

Notwithstanding the recognized limitations, the present study offers valuable information about the interface between GH and various GIC-based materials and ACAD and may guide the clinical choice of restorative materials that enhance lesion healing and limit further caries progression in primary teeth. These findings should be interpreted within the context of materials applied according to their respective clinical protocols.

## 5. Conclusions

The findings of this in vitro study highlight the impact of dentin substrate on the interface with GH and GI-based restorative materials when applied according to manufacturer instructions. The null hypothesis was rejected, as significant interface differences were demonstrated depending on the underlying dentin substrate. Microleakage of GH and GI restorative materials is higher in primary ACAD than SD. Caries-induced demineralization of hard dental tissues affects the quality of the material-dentin interface. When compared to C- and RM-GIC, GHC shows a better seal of primary dentine. From a clinical perspective, results of the present study indicate that GH restorative materials may enhance the longevity of restorations in primary teeth when selective caries removal is performed.

## Figures and Tables

**Table 1:** Preparation and application of the materials. Materials were applied according to the manufacturers’ instructions.

Material	Type	Clinical Procedure
Equia Forte HT (GC Int.)	Glass hybrid cement	Cavity conditioning: 10% polyacrylic acid (GC Dentine Conditioner, GC Int.) for 20 s, rinsing, and gentle air-drying. Capsule activation, mixing for 10 s in amalgamator, application of the material using the capsule applier. Contour shaping; initial material setting for about 2 min. Material protection: coating with Equia Forte HT Coat (GC Int.), light-curing for 20 s.
Equia Fill (GC Int.)	High-viscosity GIC	Cavity conditioning: 10% polyacrylic acid (GC Dentine Conditioner, GC Int.) for 20 s, rinsing, and gentle air-drying. Capsule activation, mixing for 10 s in amalgamator, application of the material using the capsule applier. Contour shaping; initial material setting for about 2 min. Material protection: coating with Equia Coat (GC Int.), light-curing for 20 s.
Fuji II LC (GC Int.)	Resin-modified GIC	Cavity conditioning: 10% polyacrylic acid (GC Dentine Conditioner, GC Int.) for 20 s, rinsing, and gentle air-drying. Capsule activation, mixing for 10 s in amalgamator, application of the material using the capsule applier. Light curing for 20 s. Material protection: coating with Fuji Coat LC (GC Int.), light-curing for 10 s.
Ketac Molar (3M ESPE)	High-viscosity GIC	Cavity conditioning: 20% polyacrylic acid (Ketac Conditioner, 3M ESPE) for 10 s, rinsing, and gentle air-drying. Capsule activation, mixing for 10 s in amalgamator, application of the material using the capsule applier. Contour shaping; initial material setting for about 2 min.
Photac Fill (3M ESPE)	Resin-modified GIC	Capsule activation, mixing for 10 s in amalgamator, application of the material using the capsule applier. Light curing for 20 s.

## Data Availability

The original contributions presented in this study are included in the article. Further inquiries can be directed to the corresponding authors.

## Abbreviations

The following abbreviations are used in this manuscript:

SD	Sound dentin
CAD	Caries-affected dentin
ACAD	Artificially created CAD
GIC	Glass ionomer cement
GH	Glass hybrid
HV	High-viscosity
RM	Resin-modified
EF	Equia Forte HT
E	Equia Fill
KM	Ketac Molar
F-II	Fuji II LC
PF	Photac Fill
SEM	Scanning electron microscopy
